# American Dental Association

**Published:** 1871-12

**Authors:** 


### ARTICLE III.

### *American Dental Association.*

Fourth Day—*Morning Session.*

Called to order by the President.

Resolutions instructing the Treasurer to notify members of their dues, and also instructing the Corresponding Secretary to notify standing committees of their election, were passed.

Dr. Goddard offered an amendment to the constitution similar to that rejected the day previous, allowing the time of meeting to be annually fixed. Laid over.

The Committee on Nominations made the following report, which was subsequently adopted,—and the committees so constituted:

*Dental Physiology.*—J. H. McQuillen, M. S. Dean, C. F. Wheeler.

*Dental Pathology and Surgery.*—Homer Judd, W. H. Atkinson, C. Stoddard Smith.

*Dental Histology and Microscopy*—R. W. Varney, J. Taft, W. W. Allport.

*Dental Chemistry.*—D. S. Dickerman, H. A. Smith, J. J. Birge.

*Dental Therapeutics.*—W. L. Sage, J. C. Walker, W. N. Morrison.

*Operative Dentistry.*—C. E. Francis, E. A. Bogue, Wm. Dutch.

*Mechanical Dentistry.*—I. A. Salmon, F. Hickman, J. H. Smith.

*Dental Education.*—A. B. Robbins, C. C. Chittenden, J. N. Crouse.

*Prize Essays.*—Geo. A. Mills, E. Floyd, R. Huey.

*Etiology.*—W. A. Bronson, L. D. Sheppard, G. R. Thomas.

Dr. Taft favored the appointment of a committee whose duty it should be to go at the solicitation of the profession to give clinical instruction. He thought the association ought to initiate and carry on a work of this kind. Individuals are constantly receiving invitations to go to various places, and their expenses or more are tendered. It would be better for them to go under the auspices of this society.

Dr. Dutch coincided with the views of Prof. Taft. The plan had already been proposed on the Pacific coast The expenses ought not to be borne by the association, but by the parties benefitted.

Dr. Dickerman also endorsed Prof. Taft's views. If this action is not taken by this body, it will be by some other body.

Prof. Taft then moved that a special committee on Local Societies and Conference with the Profession be appointed by this body.

Carried, and the following committee appointed:

Prof. Homer Judd, St. Louis, Mo.; Prof. J. Taft, Cincinnati, Ohio; Prof. W. H. Atkinson, New York City; Prof. L. D. Sheppard, Boston, Mass.; Dr. Wm. Dutch, San Francisco Cal.

The report of the Committee on Etiology, by Prof. Cutler, was then read by Dr. Taft. The following is a synopsis of the report.

Etiology was defined to be the science of causes; dental etiology the causes of dental diseases. Causes always precede effects. If a cause produces no effect, it is dormant. The causes of dental caries will be first considered. We quote from Tomes, the best authority extant, but we shall endeavor to point out many errors in his conclusions. Tomes says two theories have been adopted: the first considered caries as a vital action, the result of inflammation; the second regarded it as wholly the result of chemical action. Fox endeavored to show that it was similar to osseous caries, and due to a separation of the lining membrane. Bell considers that it is the result of inflammation of the dentine. Mr. Robertson assumes that it is nothing more than chemical decomposition. The opinion of Fox cannot be correct, because there is no connection between the pulp and the inner wall of the tooth—other than nerve fibres. He could have had but limited knowledge of the anatomy of teeth. Nothing could be further from the truth than Bell's ideas. Robertson assumes that tooth substance is devoid of life, which is incorrect, though this is even now maintained by able men.

Tomes thinks that dentine loses its vitality from systemic causes, and then becomes a prey to the destructive agents. We all know better at this day; a tooth decays when the vitality is intact. The process is purely local and chemical, favored, however, by crowded teeth, lodging of food in the interstices, etc., which generates acid in decomposing. There are great differences in teeth as to resisting decay, depending on the relative proportion of animal and mineral components, and on constitutional tendencies. The slower the process of development, the more dense the structure. The condition of the mother during gestation influences the teeth of the child. Subsequent systemic changes alter the condition. Bell thinks mercury is one of the remote cause of decay after the formation of the teeth. I accede to this to a certain extent. Hippocrates believed that cold caused decay; other authors, that it was due to an arrest of circulation by the pressure laterally. French writers of the last century believed in the local action of chemical agents to produce the result, as did Salmon, of London, two hundred years ago. It is said the Indians knew noth ing of bad teeth or debilitated stomachs till after the introduction of tea,—but it is more likely that the contact of civilization in general was ths cause. Dr. Mitchel demon strated, in 1796, that caries was due to an acid in the mouth. Harris says acetic, lactic, and other acids have been detected in the saliva. Donne says that the saliva is normally alkaline, but the mucous secretion acid. Harris says that Dr. Parmly, about 1821, first promulgated in this country the doctrine that decay was due to external agents. Prof. Westcott, in 1843, proved that all the acids act readily upon the teeth. Harris asks how, if caries is always due to external agents, does it sometimes commence within? I answer that it never does. Its attacks are always external; but it may be gradually going on without external sign. Tomes says all carious cavities are circumscribed by a zone of consolidated tubuli, varying in different specimens; all the tubes may be filled or only a portion of them. This is analogous to that condition in the soft parts which circumscribes inflammation by consolidation of the surrounding tissues. It retards the advance of decomposition and protects the pulp from irritation. It is like an army falling back gradually, constructing lines of defense and holding each as long as possible. I am not able to see the point in his comparisons. It would appear that he regards the tubuli as unoccupied channels through the dentine, which I am not prepared to admit; if it were true, what causes sensitiveness? The tubuli are, no doubt, filled up to a certain extent, but the material is necessarily derived from the secretions of the mouth becoming hard by dessiccation, and like tartar preservative of the dentine. I regard the process as a molecular one, independent of vitality.

When decay attacks dentine, there is an invasion into vital and mineral tissues which have before been under vital control alone, by vital and chemical agencies combined. When decay is established, the part is under a chemical control alone; it overcomes all resistance by satisfying affinities that are stronger than the vital force. It is true that the greatest density affords the greatest resistance to decay; but want of density signifies imperfect development; as the animal per cent. is about the same in all. Prof McLain says the chemical theory being now generally admitted, the physico-defective theory can be considered only as one of the proximate causes. Imperfections favor decomposition only by affording lodgment to foreign matters which are converted into an acid product; or acids already formed may be taken in; which, often repeated, produces decay. I fully concur in these views. Maury says authors have never agreed as to the cause of caries; some have regarded it as hereditary; others as necrosis; and others still as among the ulcerous affections. We believe it is due to external and internal causes; external being blows, contact of cold and hot substances, or acid drinks; malformation, lateral pressure, malaria, mercury, uncleanliness, and irregularities. He enumerates internal causes, which have already been mentioned. Fitch says a vitiated state of the saliva is the first general cause. Lead, tin and copper are soon dissolved in it, and gold when scarcely acted on by nitric acid is tarnished; ivory soon decays; and it is not strange that teeth are also acted upon perniciously. Uncleanliness vitiates this fluid; decaying food, decayed teeth or roots, etc. In summing up, we say that decay of the teeth is dependent on violations of physiological law. Some of the foregoing opinions are erroneous, owing to the fact that there has been no established canon for the profession to be governed by; every one had his peculiar views. The most modern, and in the opinion of the committee the nearest true, if not the true cause of caries, is the chemical theory of an acid action as the only direct local action. The effect is the same, however the acid may get into the mouth, depending, however, upon the condition of the mucous and digestive appendages. Modern civilization gives evidences of violated law, which not only causes decay of the dental organs, but also other pathological conditions which are suicidal, by shortening the natural term of human life.

The report of the Committee on Operative Dentistry was then called for, and was read by Prof. Taft.

The report proposed merely a statement of a few points. That of last year was very comprehensive, embracing all points of interest up to that time. Progress is being made, not alone of a mechanical character, but there is a growing domination of science over art, of principles over manipulation. Though there are no startling facts in reference to modes of procedure in operating, forward steps are being made.

There is a far greater disposition to adopt new processes, which is instanced in the increasing use of the rubber dam, a knowledge of the use of which carries with it a conviction of its utility, and usually its immediate adoption.

There is a growing disposition to use great care and thoroughness in the operation of filling. The object is not attained without a careful attention to every step; an imperfect link involves a broken chain. We suggest that the profession strive to come up to attainments already made.

The use of glasses is recommended, as tending to reveal imperfectons and irregularities which by the unaided vision will escape observation. Their discriminating use will be of benefit to every one, and will not be injurious to the unimpaired eye, as is often supposed.

In the use of foil some change has been suggested, viz., the use of smooth and polished points for its introduction. It is claimed that these may be substituted for serrations in all cases, even in building up crowns, and that the results are much more perfect. However this may be, it is quite certain that by the proper use of such points, foil may be thoroughly welded. It is claimed that with them the gold will expand more, and thus be more perfectly adapted to the walls, with less liability of injury to the latter. A frequent cause of failure is the injury done to the margins of cavities. The occasional use of these points was adopted by a few persons two or three years ago, but their exclusive use has been only for a few months. A claim to this use has been made by three or four persons, but no one has been so enthusiastic in their favor as Dr. A. A. Blount of Springfield, Ohio. The introduction of a change like this will not be rapid, even though it is found to be a step in advance. The use and proper place of heavy foils is more definitely ascertained than at the time of the last report. A few use them exclusively, but the majority employ them after the cavity has been chiefly filled, to form the surfaces and borders, for which purpose they are better adapted than anything else. It is stated that No. 480 can be used; its practicability to any extent, however, is doubtful, although from six to ten thicknesses of No. 60 can be easily and thoroughly welded,—but the laminæ must be kept free from folds.

For non-metallic fillings, Guillois's cement seems to possess greater hardness than any of the kindred preparations, and many operators employ it as a permanent filling. Its efficiency for this purpose, however, is not sufficiently demonstrated,—but as a foundation for a gold filling it is very valuable. It is probably allied to oxychloride of ziuce.

The machines for excavating cavities and finishing fillings have been much improved since the last meetings when their utility was a matter of doubt. Now, in two of them such is the perfection and efficiency, that a first view carries conviction of their usefulness, and they are now regarded as indispensable to the most thorough and rapid execution of the operation of filling. Improvement is taking place in every department of human activity, and we cannot venture to assert what is in store for us, or what shall be brought to us upon the great tidal wave of progress. We can, however, admire, appreciate, use and rejoice in all the blessings already bestowed upon us.

Dr. Atkinson. If the concepts of the mind in attempting to understand our practices are foundlings, we are much in the dark.

Prof. Judd. I have experimented with smooth points, and cannot work quite as well with them as with points slightly serrated, because if the instrument is not placed perpendicular it is a little apt to slip. If it is a sharp-edged instrument, the same office is performed by the serrations. I have used planishing points, and can work very well with them. I have used platinum foil a little. That used was heavier than ordinary gold foil. It is coated with gold foil to secure the adhesive property. A filling made of it looks like a gold filling before finishing, but after finishing presents a mottled appearance.

Dr. Atkinson. I endeavored to get platinum foil beaten in 1845, but never succeeded until within a year. I thought that platinum, osmium, or some of that group ought to be capable of use in this way. I still feel that if we have pure platinum rolled, it will weld. I have used a thickness corresponding to No. 120, which makes the best filling. As to points, I use the egg-shaped planishing points. Welding is the annihilation of space between particles. If we understood the laws of impact and welding, we need say nothing about these matters. Not one per cent. of practitioners understand the proper preparation of cavities—to prepare the cavity so as not to expose the pulp; then so that the gold may be mechanically retained. Don't be afraid of cutting too much. I don't cut enough myself, and I am called heroic. Little fissures are too often lent. Cut freely and largely, and don't do any of this "*that'll do*" kind of work. Do not remove the decayed bone and expose the pulp. Nature has built that house; don't pull it down. Cut to solid tissues around the wall,—saturate with thymol, which says, Peace, be still, to all the little devils in there!

Dr. McQuillen spoke of the importance of a thorough examination of the teeth. It cannot be done with the naked eye, but demands the employment of magnifying-glasses. The professional man should be paid for his opinion, which it costs him time to give. A microscopical opening may lead to an extensive cavity below, and he would impress the necessity of using magnifying-glasses. He spoke also of the matrices proposed by Dr. L. Jack for approximal fillings as of great value; simplifying the operations, and rendering success in such cases more certain than without them.

Dr. Taft referred to the use of glasses upon the eye. He used two pair of glasses at once, but as a substitute had constructed a pair with a lens attached at one and a half inches distance; is much at a loss without it. It is common to suppose that tne use of glasses impairs the eye, but our best oculists concur that there is no objection to the use of glasses on the unimpaired eye. The microscpe may be used for a lifetime without detriment—the eye-glasses of the watch maker are so used. The sight has been improved in his own case in the eye to which this glass has been fitted. There is a want of precision in trimming the borders of cavities without glasses. They should be used constantly.

Dr. Francis. We cannot make our examination thorough without glasses. I always advocate cutting out fine fissures. It is dangerous practice to cut to the pulps. What better covering can we have than the natural one?

Dr. Crouse does not lay quite as much stress on glasses as some do whose sight has partially failed. Thorough examinations are necessary, and the rubber dam should be applied for this purpose; also, free separation should be made by wedging, so as to be certain in regard to decay.

Dr. Morgan endorses all that has been said in reference to thorough examinations, also as to cutting out fissures, especially in molars; has used the magnifying-glass for many years. It is fair to presume that partially decayed matter is not dead; it is certainly tolerated. Heavy foils can be planished down very perfectly.

Dr. McQuillen. It is impossible for any one to see small objects with the naked eye as with a magnifying glass. Engravers have used such glasses for ages.

Dr. Francis endorses what has been said in reference to the dam. Glasses include mouth mirrors, and even these he believes are not often used. He spoke of a cavity which externally gave no signs of its presence, except that there was a slight seam on the grinding surface, and the tooth was a little opaque, which being investigated, led to an immense cavity approaching the pulp.

Dr. Atkinson. No human eye unaided has yet detected tissues, or any of their individual elements.

Dr. Dickerman used smooth points four years since with little pressure, and latterly fills mostly in that way, and makes better fillings than with so much pressure or with the mallet.

Dr. Dutch claims that some persons have better eyesight than others; mentioned a gentleman who could see fine cracks in the enamel with the naked eye, where only a glass of high power revealed them to others. He cuts out fissures well.

Dr. McDonneld endorses all that has been said; has not yet arrived near perfection in making examinations; endorses the dam and magnifying-glasses fully. Fissures can be discovered with the glass which cannot with the eye alone—even with the best eyes. We are not thorough enough in cutting down weak walls and following out fissures.

Dr. Walker thinks we come to the conclusion every year that last year we did not know how to fill a tooth. He would not continue practice without the dam. The power of the best eyes can be extended by glasses. Fissures will often lead to an unsuspected chamber.

Dr. Salmon. Any one who acknowledges the use of the microscope will acknowledge the use of glasses. He mentioned some one who is in the habit of using ivory points, and commends trying the method in crown cavities which are accessible.

Dr. Crouse explained that he thought in many cases glasses were desirable. When you can't see with the naked eye, be sure to use them.

Dr. McDonneld spoke of the use of the rubber dam. We should not condemn it for want of skill in its use. He had failed, and abandoned it, but afterwards tried again and succeeded.

Dr. Judd called attention to the word approximal which is often used. There is no such word.

The Committee on Mechanical Dentistry was then called and Dr. W. N. Morrison made a report.

The report stated that the committee were unable at the time to present anything of practical benefit which had not already been noticed in the dental journals. The different styles of work stand in the same order that they have for years, continuous gum and gold being at the head of the list. The so-called cheap materials, rubber, celluloid base, pyroxyline, porcelain base, aluminum, and the alloys of tin, have each their advocates, but upon the superiority of either we are unable to agree. The committee were inclined to believe that the demand for these cheap bases is occasioned by the want of teeth in proper variety and form for gold work

Dr. Walker, from the same committee, recommended Watt & Williams' metal as the best substitute for rubber that we have now. Had not tried collodion.

Dr. Floyd, from the same committee, prefers gold to everything else, uses a little rubber in temporary cases; cannot consider it equal to gold; has used the cheoplastic metal, but does not approve of alloys.

Dr. McDonneld found that black rubber would cure a congested condition of the mouth. Gold and continuous gum are the best; cheap things are dear.

The Committeee on Dental Education was then called, and Dr. Atkinson read a report from Dr. Mills, Chairman.

The report stated that every avenue was teeming with the spirit of progress; the associated effort has done a noble work, not so much in the older localities as over the whole land. How shall we best extend the resources of dental education? We have a right to expect much of those who stand out as representative dental educators. Gentlemen, speak out of your convictions, the fruit of experience and of toil. Tell us, must we go deeper, ere we can safely go higher? Do we need to divide, regulate, separate, that we may better understand our work? What is our mission? Do we answer, as one man, it is a God-given, a God-loving one? Or do some among us call it a mission of dollars and cents? Our mission is of God. We are to repair the ravages of this disintegrated structure; shall we attempt to repair without knowledge of that structure? Tell us what we need to understand, that we may think and know for ourselves. The associate element has proved itself the great auxillary in dental education; it brings us into a receptive condition; mellows the soil of our nature, and lets in the sunlight of brotherly love. Association is to us as the plow in the tilling of the soil. May those attending this body go out to be known and read of all men, that they have been with truth and learned of it. The rudimental teachings have been and will be received in the dental office. No dentist or faculty should encourage an applicant to enter a dental school before spending a year or more under private teaching. The dental schools are doing a noble work; they show an increased tendency to meet the demands of the times. Whether they are meeting these demands is a question seriously agitating the minds of many. A combination of the best ability of our profession should now be made, and this United States be the proud possessor of an American dental university—not to substitute the dental schools, but to meet the higher demand that is upon us. Would to God that this noble profession might be the first to throw out to the millions the motto—"Obedience to the truth."

Strip us of our meaningless titles, attacked so freely; because of the freedom with which they are dispensed they are become a stench to all true lovers of the truth. These words, spoken in sorrow, are the truth. With all the worth of the schools, they are terribly deficient. The responsibility must rest chiefly on the faculties; they have the power to reject unfit applicants. It were better to lessen the quantity and better the quality. Furnish first-class teach ings, and you will secure first-class pupils. Schools need encouragement, and also to be shorn of much. We see much to admire, and much to regret; some things are clear, much is misty. Yet in view of all their deficiences, if we turn to the past, the comparison renews our spirit. But a few years back there could be found in New York dentists who denied to their students the use of books; and in one instance, a work in the possession of a student was obtained by theft and committed to the flames. And this not among quacks or "gutter dentists," but among those who walked in high places. Education teaches knowledge of obedience to law. We are groaning under the penalty of a broken law. We are crawling backwards to reach the bottom of the ladder. It requires courage to go down, and also to go up. Let us strip ourselves of old dogmas, and search only for the truth. Those who apply for admittance must be impressed with the necessity for certain qualifications. There are found in all our schools those who possess hardly a scintilla of the requisites in the specialty they are seeking—some have not the first teachings of the common branches. Ask the classes admitted each year what they seek, and nine-tenths of them will say "a business." Our sense of need must first be felt. That inspiration is the beginning of all knowledge, is as true as that God exists. We will find the bottom if we let go. If the descent is rapid, the return to light will be so too. We shall bemean ourselves if we seek for anything less than the highest and best. Some may ask what this has to do with dentistry; some, too, accept these standards and nothing less. The signs of the times indicate all that the pnrest desire can wish. The nations are crying out as much for prevention as for cure. As we are more fully educated in the principles, we more fully understand the preventive remedies. The dentist must be educated to be more than the so-called dentist; he must be a dental physician—able to deal with all the conditions of both health and disease. Nothing short of a medical as well as dental education is the demand of the hour. That there will be differences among us is not to be wondered at; but it is possible that we be of one spirit—"whereto we have attained, let us walk."

Of our journals, it is far from the truth to say that they are what they should be; yet we should be grateful that they are as much credit to the profession as they are. The periscopic method is on the increase with all the journals, with a decided advantage to the readers. All who have ability should feel it a pleasure to contribute to them, without fear of being rejected. The dentists who associate are readers; none can read the journals without profit.

Instruction by clinics has, during the year, showed marked increase. The associations have observed them at their gatherings with profit; the schools have given them increased attention; and, as a new feature, public clinics are held by the First District Dental Society of New York, under the direction of a committee, in the rooms of S. S. White's Dental Depot, each Wednesday. A record of all operations is kept and reported to the society. The attendance of a large number of dentists from all parts of this and other countries has been secured. The Brooklyn Dental Infirmary has been established by the Brooklyn Society, and is believed to be the first and only one of the kind. Clinics are also held here each week. It has grown to its present proportions at no little sacrifice of time and money, but those who have participated have reaped a rich harvest. The best method of reaching the public has yet to be determined. Some individuals have made efforts,—with how much encouragement is yet to be made known. Let us impart in a true spirit of liberality, and let our communications be yea, yea, while in the light, and nay, nay, when darkness presents itself. So do, and further generations will rise up and call us blessed.

(*To be Continued.*)

